# Safety and Immunological Evaluation of Interleukin-21 Plus Anti-α4β7 mAb Combination Therapy in Rhesus Macaques

**DOI:** 10.3389/fimmu.2020.01275

**Published:** 2020-07-17

**Authors:** Maria Pino, Srijayaprakash Babu Uppada, Kabita Pandey, Colin King, Kevin Nguyen, Inbo Shim, Kenneth Rogers, Francois Villinger, Mirko Paiardini, Siddappa N. Byrareddy

**Affiliations:** ^1^Division of Microbiology and Immunology, Yerkes National Primate Research Center, Emory University, Atlanta, GA, United States; ^2^Department of Pharmacology and Experimental Neuroscience, University of Nebraska Medical Center, Omaha, NE, United States; ^3^New Iberia Research Center, University of Louisiana at Lafayette, New Iberia, LA, United States; ^4^Department of Pathology and Laboratory Medicine, Emory University School of Medicine, Atlanta, GA, United States; ^5^Department of Genetics, Cell Biology and Anatomy, University of Nebraska Medical Center, Omaha, NE, United States; ^6^Department of Biochemistry and Molecular Biology, University of Nebraska Medical Center, Omaha, NE, United States

**Keywords:** anti-α4β7, IL-21, immune activation, T- cell homing, macaques, rhesus macaques, combined immune intervention

## Abstract

Human immunodeficiency virus (HIV) and simian immunodeficiency virus (SIV) infections compromise gut immunological barriers, inducing high levels of inflammation and a severe depletion of intestinal CD4^+^ T cells. Expression of α4β7 integrin promotes homing of activated T cells to intestinal sites where they become preferentially infected; blockade of α4β7 with an anti-α4β7 monoclonal antibody (mAb) prior to infection has been reported to reduce gut SIV viremia in rhesus macaques (RMs). Interleukin-21 (IL-21) administration in antiretroviral therapy-treated, SIV-infected RMs reduces gut inflammation and improves gut integrity. We therefore hypothesized that the combination of IL-21 and anti-α4β7 mAb therapies could synergize to reduce inflammation and HIV persistence. We co-administered two intravenous doses of rhesus anti-α4β7 mAb (50 mg/kg) combined with seven weekly subcutaneous infusions of IL-21–IgFc (100 μg/kg) in four healthy, SIV-uninfected RMs to evaluate the safety and immunological profiles of this intervention in blood and gut. Co-administration of IL-21 and anti-α4β7 mAb showed no toxicity at the given dosages as assessed by multiple hematological and chemical parameters and did not alter the bioavailability of the therapeutics or result in the generation of antibodies against the anti-α4β7 mAb or IL-21–IgFc. Upon treatment, the frequency of CD4 memory T cells expressing β7 increased in blood and decreased in gut, consistent with an inhibition of activated CD4 T-cell homing to the gut. Furthermore, the frequency of T cells expressing proliferation and immune activation markers decreased in blood and, more profoundly, in gut. The combined IL-21 plus anti-α4β7 mAb therapy is well-tolerated in SIV-uninfected RMs and reduces the gut homing of α4β7^+^ CD4 T cells as well as the levels of gut immune activation.

## Introduction

Human immunodeficiency virus (HIV) infection induces high and persistent levels of immune activation and inflammation, which are associated with the loss of CD4^+^ T cells and accelerated disease progression (1, 2). With the advances in antiretroviral therapy (ART), the incidence of HIV infection and transmission has been reduced significantly. However, despite effective viral suppression in plasma, ART does not cure HIV infection, with virus persisting in long-lived CD4^+^ T cells or macrophages in different tissues and organs (3). Furthermore, ART-treated HIV-infected individuals can still present persistent chronic inflammation, limited CD4^+^ T-cell reconstitution, and mucosal immune dysfunction (1, 4–6), which have all been linked to increased HIV- and non–HIV-associated comorbidities and mortality. Therefore, new therapeutic strategies aimed at reducing both viral reservoir and chronic immune activation in combination with ART could be beneficial for a potential cure strategy.

Interleukin-21 (IL-21) is a pleiotropic cytokine, member of the common ɤ-chain-signaling family, which includes IL-2, IL-4, IL-7, IL-9, and IL-15, and it is mainly produced by CD4 T helper (T_H_) cells (including T_H_17 and Tfh), ɤδ T cells, CD8 T, and natural killer (NK) T cells. Interleukin-21 affects multiple pathways of both humoral and cell-mediated immune responses (7). Previously, we showed that in simian immunodeficiency virus (SIV)-infected rhesus macaques (RMs) loss of IL-21–producing CD4^+^ T cells in the gut is associated with T_H_17 cell depletion, loss of gut mucosa integrity, and mucosal immune dysfunction (8). Moreover, we have shown that administration of a rhesus IL-21–IgFc fusion protein in acute (9) or chronic ART-treated SIV-infected RMs (10) resulted in the preservation of intestinal T_H_17 cells, improved mucosal immune function, and reduced microbial translocation. Finally, we also showed that IL-21 treatment resulted in a reduction of the replication competent viral reservoir in lymph nodes (10). Importantly, a cross-sectional human study showed that IL-21 production is decreased at the very early stage of HIV infection and that serum IL-21 concentrations correlate with CD4^+^ T-cell counts (11). In contrast, normal levels of IL-21–producing CD4^+^ T cells were observed in HIV elite controllers, individuals able to naturally (without ART) control HIV replication to very low levels (11). Furthermore, it has been shown that IL-21 promotes degranulation and effector functions of CD8^+^ T cells (12, 13) and that IL-21–producing HIV-1–specific CD8^+^ T cells are more abundant in elite controllers (14).

α4β7 integrin is a key molecule for mucosal homing of lymphocytes (15), and α4β7^+^ CD4 T cells, including T_H_17 cells, are the primary targets and thus rapidly depleted during the initial phase of HIV and SIV infection (2, 16, 17). Previous studies in RMs suggest that α4β7 blockade could limit the number of activated and preferentially infected cells to gastrointestinal-associated lymphoid tissues (GALTs), with the potential to reduce both viral loads and chronic inflammation within the gut. Treatment with a primatized anti-α4β7 monoclonal antibody (mAb) initiated prior to SIV infection in RMs has been shown to reduce mucosal transmission and reduce the viral loads within the gut (18).

Collectively, data generated with these single interventions showed that α4β7 blockade limited viremia in mucosal sites of HIV persistence, and IL-21 promoted the reconstitution of mucosal T_H_17 cells, critical to maintain mucosal integrity and limit microbial translocation, one key cause of chronic immune activation in HIV and SIV infection. Therefore, we propose that a combined strategy based on administration of IL-21 and anti-α4β7 mAb could have the potential to limit inflammation and, as a consequence, improve antiviral immune responses and reduce viral persistence in ART-suppressed HIV-infected individuals. Although IL-21 and anti-α4β7 mAb administration has been tested individually and found to be safe, co-administration of the two compounds has never been tested or reported. Here, we conducted a pilot study aimed at determining the safety, tolerability, and biological activity of the combined IL-21 and anti-α4β7 mAb treatment in healthy, SIV-uninfected RMs. The data generated from this pilot study will guide future combined interventions in ART-treated SIV-infected non-human primates, aimed at limiting residual inflammation and viral persistence.

## Materials and Methods

### Animal Ethical Consideration and Treatment

All animal experiments were conducted following guidelines established by the Animal Welfare Act and the National Institutes of Health (NIH) for Housing and Care of Laboratory Animals and performed in accordance with institutional regulations after review and approval by the Institutional Animal Care and Usage Committees at the Yerkes National Primate Research Center (YNPRC). Anesthesia was administered prior to performing any procedure, and proper steps were taken to minimize the suffering of the animals in this study. A total of four Indian origin RMs (*Macaca mulatta*) were enrolled in this pilot study (Supplementary Table 1). All macaques were housed and maintained at the YNPRC (Atlanta, GA, USA). All animals received two doses of rhesus anti-α4β7 mAb (50 mg/kg, intravenous route) obtained from NIH Non-human Primate Reagent Resource, University of Massachusetts Medical School, Worcester, MA, USA, at 3-week interval (days 0 and 21) and seven weekly doses (days 0, 7, 14, 21, 28, 35, and 42) of recombinant rhesus IL-21–IgFc (IL-21-Fc, 100 μg/kg, subcutaneous route) obtained from Resource for Nonhuman Primate Immune Reagents of the New Iberia Research Center.

### Sample Collection and Processing

Blood and rectal biopsies (RBs) were collected at multiple time points before, during, and after the interventions. Blood samples were used for complete blood counts and comprehensive serum chemistry panels. Plasma was separated from EDTA-anticoagulated blood by centrifugation within 1 h of phlebotomy. Density centrifugation was used to isolate peripheral blood mononuclear cells (PBMCs). Up to 20 RBs were collected with biopsy forceps under visual control via an anoscope. Rectal biopsy–derived lymphocytes were isolated by digestion with 1 mg/mL collagenase for 2 h at 37°C and then passed through a 70-μm cell strainer to remove residual tissue fragments. All samples were processed, stained, fixed (1% paraformaldehyde), and analyzed by flow cytometry within 24 h of collection as described previously (10).

### Flow Cytometric Analysis

Flow cytometric analysis was performed on PBMCs and RB-derived cells according to standard procedures using a panel of mAbs that others and we have shown to be cross-reactive with RM immune cells (10, 19) (Supplementary Table 2). The following Abs were used: anti–CD4-APCCy7 (clone OKT4), anti–HLA-DR-BV711 (clone L243), and anti-CD20 PerCpCy5.5 (clone 2H7) all from Biolegend, San Diego, CA, USA; anti–CD95-CF594 (clone DX2), anti–beta7-PECy5 (clone FIB504), anti–CCR7-PECy7 (clone 3D12), anti–Ki67-Alexa700 (clone B56), anti–CD3-BUV395 (clone SP34-2), anti–CD8-BUV496 (clone RPA-T8), anti–CD56-BV605 (clone B159), and anti–CD16-BV650 (clone 3G8) all from Becton–Dickinson, BD Biosciences, San Jose, CA, USA; anti–NKG2A-APC (clone Z199), from Beckman Coulter, Brea, CA, USA; Aqua Live/Dead amine dye-AmCyan from ThermoFisher Scientific, Invitrogen, Waltham, MA, USA; anti–CD38-FITC (clone AT-1) from STEMCELL Technologies, Vancouver, British Columbia, Canada; and anti–α4β7-PE (clone Act-1) obtained from the NIH Non-human Primate Reagent Resource, University of Massachusetts Medical School. Flow cytometric acquisition was performed on at least 100,000 CD3^+^ T cells on a BD LSRII Flow Cytometer driven by BD FACSDiva software. Analyses of the acquired data were performed by FlowJo software, Tree Star, Inc., Ashland, OR, USA.

### Measurement of Rhesus Anti-α4β7 mAb in Plasma

Levels of rhesus anti-α4β7 mAb in plasma samples from the four macaques were quantified as previously described (20). Briefly, HuT78 cells were first incubated at 37°C for 3 days in RPMI 1640 media containing 1 μM retinoic acid to increase surface expression of α4β7; 1 × 10^5^ cells/well were dispensed into 96-well plates and incubated with plasma (1:10, diluted in phosphate-buffered saline (PBS)/2% fetal bovine serum) for 30 min at 4°C. Cells in the wells were washed and incubated with biotinylated antirhesus IgG1 kappa (clone 7H11; NIH Non-human Primate Reagent Resource, University of Massachusetts Medical School) for 30 min at 4°C and then washed again and resuspended in neutravidin-PE (A-2660; ThermoFisher Scientific) for 20 min at 4°C. Cells were washed, fixed in 2% paraformaldehyde, and analyzed on a flow cytometer (Attune NxT; ThermoFisher Scientific). Rhesus anti-α4β7 antibody was quantified using a standard curve method by comparing the mean channel fluorescence intensity mean channel fluorescent intensity (MFI) of cells treated with macaque plasma to the mean channel fluorescence intensity MFI of cells treated with serially diluted rhesus anti-α4β7 mAb (clone Act-1, obtained from NIH Non-human Primate Reagent Resource, University of Massachusetts Medical School).

### Measurement of Rhesus Anti-rhesus (Anti-drug) Antibodies

To determine whether the rhesus may potentially generate antibodies against the infused recombinant rhesus anti-a4b7 mAb, an enzyme-linked immunosorbent assay (ELISA)–based assay was developed to monitor the detection of such rhesus anti-rhesus Ig antibodies (RARA), also named anti-drug antibodies (ADAs). Detection of antibodies generated against rhesus IgG1 kappa chain in RM plasma was measured by detecting monoclonal anti-lambda light chain bound to immobilized rhesus recombinant anti-α4β7 antibody by ELISA assays. In brief, ELISA plates (ThermoFisher Scientific) were coated with anti-α4β7 antibody (NIH NHP RR–Rhesus Recombinant IgG1 kappa, CDR-g, lot no. 092012G) in coating buffer (1 × PBS) at 10 μg/mL. 100 μl were added to individual wells of the 96-well microtiter plate and left overnight at 4°C. Plates were then washed six times with wash buffer (PBS/0.05% Tween 20) and blocked with 300 μL per well of Superblock solution (ThermoFisher Scientific) for 15 min at room temperature (RT) followed by washing six additional times with wash buffer. The test sera from the monkeys to be screened for ADAs were 4-fold diluted (starting at 1:10) in dilution buffer (PBS/2% bovine serum albumin) and dispensed into duplicate wells at 100 μL per well. After 1-h incubation at RT, plates were washed six times with wash buffer followed by the addition of 100 μL of a 1:100 dilution of a monoclonal anti–lambda light chain-biotin (clone IS7-24C7; Miltenyi Biotech, Cologne, Germany) per well. After 1-h incubation at RT, plates were washed six times with wash buffer followed by the addition of 100 μL of a 1:10,000 dilution of streptavidin–horseradish peroxidase (HRP) (Invitrogen) per well. After 1-h incubation at RT, wells were washed six times with wash buffer followed by the addition of TMB substrate (SeraCare, Gaithersburg, MD, USA) at 100 μL per well to develop color and finally halted with stop solution containing H_2_SO_4_ (KPL). The optical density (OD) was recorded at 450 nm on Spectramax i3x plate reader (Molecular Devices, San Jose, CA, USA). Controls consisted of wells with baseline monkey sera and PBS alone (negative control); sera from a previously titered ADAs containing positive sera served as a positive control. Briefly, the positive control RNo13 was a RM infected intravenously with SIV_mac239_, which initiated ART at week 5 of infection for a 90-day course. At week 9 of infection, and similarly to our study, this animal received 50 mg/kg of mAb against α4β7 intravenously every 3 weeks (21). The end point was noted as the highest dilution of the test sera with OD >2 × pretreatment sample, and this dilution was considered positive for the assay.

### Measurement of Rhesus IL-21–Fc in Plasma

Maxisorp 96-well plates were precoated overnight at 4°C with 2 μg/mL purified anti–human IL-21 capture mAb (clone J148-1134; BD Biosciences) in 100 μL bicarbonate buffer pH 9.6 per well. The next morning, the unbound antibody was removed, and the coated plates were blocked for 2 h with 300 μL per well of PBS with 2% bovine serum albumin at 37°C. The plates were then washed four times with PBS supplemented with 0.05% Tween 20, added serial 2-fold dilutions of test plasma samples in duplicates and a dilution series of a IL-21–Fc standard, and incubated for 2 h at RT. Plates were washed and added 100 μL of anti–IL-21–biotin detection mAb (clone I76-539; BD Biosciences) at a 1:2,000 dilution and incubated for 2 h at RT. After washing, the plates were added HRP-conjugated Avidin D (Vector Laboratories, Burlingame, CA, USA) at a 1:2,000 dilution followed by TMB substrate (KPL) in sequential steps. The reaction was stopped by the addition of 20 μL of 1 M H_2_SO_4_, and the absorbance read at 450 nm using a Bio-Tek Synergy HT multimode microplate reader. Baseline plasma samples for each test subject collected prior to IL-21 administration were included to determine background values. The lower detection limit of IL-21–Fc was 15.6 pg/mL.

### Measurement of Rhesus Anti–IL-21–Fc Antibodies

To determine whether the rhesus may potentially generate antibodies against the infused recombinant rhesus IL-21–Fc, an ELISA-based assay was performed. Maxisorp 96-well plates were precoated overnight at 4°C with 4 μg/mL recombinant rhesus IL-21–Fc in 100 μL of 1 × coating solution (KPL) per well. The next morning, plates were washed with PBS 0.05% Tween-20 and blocked by adding 200 μL/well of PBS with 1% bovine serum albumin (blocking buffer) at 4°C overnight. After washing the plates, 100 μL plasma samples diluted (1:100, 1:1,000, and 1:10,000) in blocking buffer were added in duplicate to wells, or blocking buffer was added to wells for negative and positive control wells. Following an incubation at 4°C overnight, the plates were washed, and 100 μL of monkey cross-reactive goat anti–human kappa-Biot (Southern Biotech, Birmingham, AL, USA) diluted 1:1,000 in blocking buffer was added to sample and negative control wells. To positive control wells, 100 μL of goat anti–monkey IgG biotin (Rockland, Limerick, PA, USA) diluted 1:1,000 in blocking buffer was added. The plate was incubated for 2 h at RT. After washing, HRP-conjugated avidin D at a 1:1,000 dilution was added to wells and incubated for 1 h. Plates were washed and developed with TMB substrate and read on a microplate reader as described above for the IL-21–Fc capture ELISA. Baseline plasma samples for each test subject collected prior to IL-21 administration were included to determine background values. The positive control confirmed the correct coating of the wells and consisted of a mouse anti–human IL-21 mAb (clone I76-539, BD #558502) that cross-reacts with the rhesus IL-21–Fc, and it specifically binds to this cytokine. Of note, with this assay, we do not detect the lambda chain antibodies, but across multiple isotypes (IgA, IgG, IgM).

### Statistical Analysis

Data analyses were performed using GraphPad Prism (GraphPad Software, Inc., La Jolla, CA, USA). The results are expressed as the mean ± SD. Statistical significance (*P*-value) of immunophenotyping data between time points was not reported because of the limited number of animals included in the pilot study.

## Results

### The Combined Administration of IL-21 and Anti-α4β7 mAb Is Safe and Tolerated in RMs

**Figure 1:**
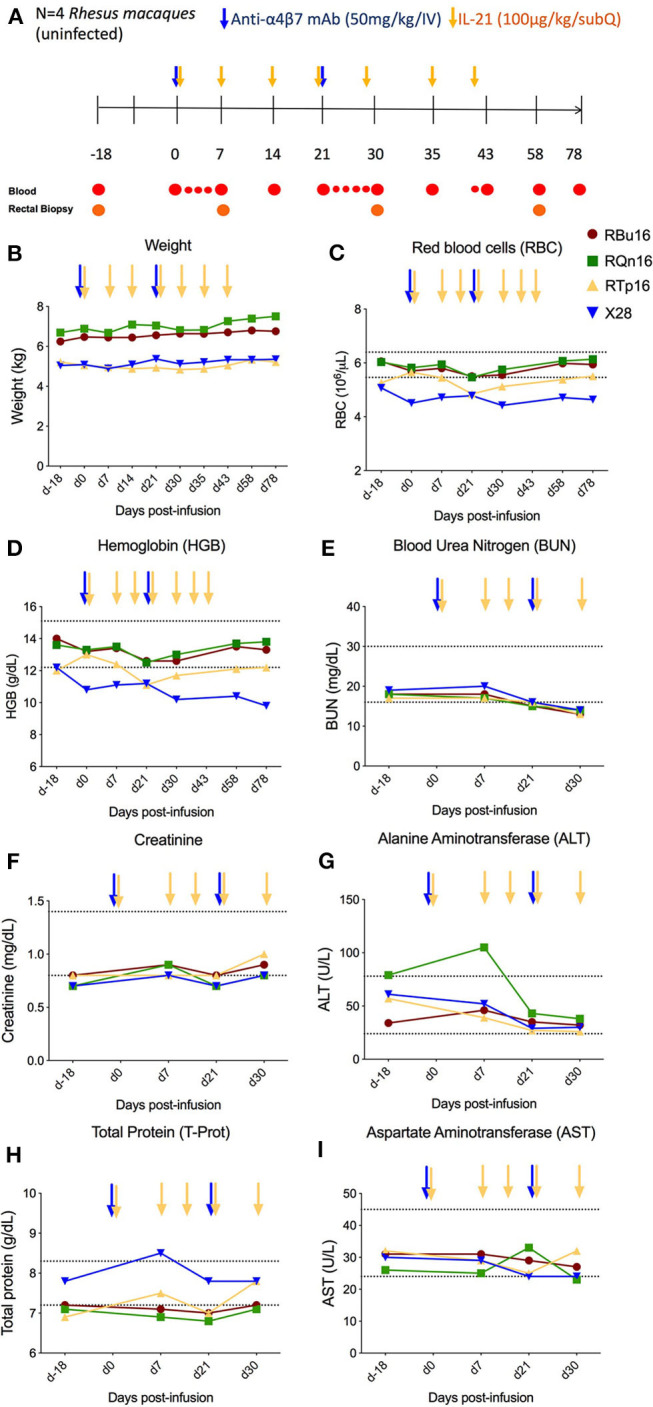
Co-administration of anti-α4β7 mAb and IL-21. **(A)** Schematic study design of co-administration of anti-α4β7 mAb and IL-21 in uninfected RMs (*n* = 4). Prior to treatment, baseline blood and RBs were collected. On day 0, 50 mg/kg of anti-α4β7 mAb was administered intravenously along with 100 μg/kg of IL-21 subcutaneously. IL-21 was given weekly up to 6 weeks (day 42 post-infusion). 50 mg/kg of anti-α4β7 mAb was infused in two doses on day 0, week 3 (day 21 post-infusion). Blood samples were collected at regular intervals as shown in the schema. **(B)** Weight changes were recorded regularly until the end of treatment. **(C)** Red blood cells, **(D)** hemoglobin, **(E)** BUN, **(F)** creatinine, **(G)** ALT, **(H)** total protein, and **(I)** AST were analyzed from the blood collected at regular intervals until the end of the study. Individual animals are represented with different colors and symbols. Baseline days are indicated as d-18 and d0. Normal range levels of each parameter analyzed are indicated in dashed lines. Blue arrows indicate the anti-α4β7 mAb intravenous infusions, and yellow arrows indicate IL-21 subcutaneous infusions.

IL-21 and anti-α4β7 mAbs have been previously administered as single interventions in naive or SIV-infected RMs with an acceptable safety profile (10, 18, 21–25). However, combined administration of the two reagents has not yet been tested. To determine the safety and tolerability of the combined IL-21 and anti-α4β7 mAb administration in non-human primates, four healthy, SIV-uninfected RMs were treated with two doses of anti-α4β7 mAb (50 mg/kg, intravenous) at a 3-week interval (days 0 and 21) and seven weekly doses (from days 0 to 42) of recombinant rhesus IL-21–IgFc (IL-21-Fc, 100 μg/kg, subcutaneous) (see section Materials and Methods and Figure 1A). First, we measured variations in weight and multiple hematological parameters. All four RMs included in the study showed stable or increased weights up to day 78 post-infusion of the combined treatment, the latest assessed experimental point, when compared with pre-treatment baseline (Figure 1B). Multiple hematological parameters were analyzed to determine possible anemias [red blood cell (RBC) count and hemoglobin (HGB)] or kidney dysfunction [blood urea nitrogen (BUN)]. We did not find any significant changes in RBC, HGB, and BUN levels, which remained stable over the entire follow-up period (Figures 1C–E). Then, we monitored serum chemistry parameters such as creatinine (kidney function), alanine aminotransferase (ALT; liver function), total protein (T-Prot; kidney and liver functions), and aspartate aminotransferase (AST; kidney, liver, and heart function) and found no significant variation from baseline with all measured values for the entire follow-up period (Figures 1F–I). Thus, at the doses administered, combined administration of IL-21 and anti-α4β7 mAb is well-tolerated and has no detectable toxic effects.

### Co-administration of IL-21 and Anti-α4β7 mAb Does Not Induce ADAs Or Alter the Bioavailability of the Two Compounds

**Figure 2:**
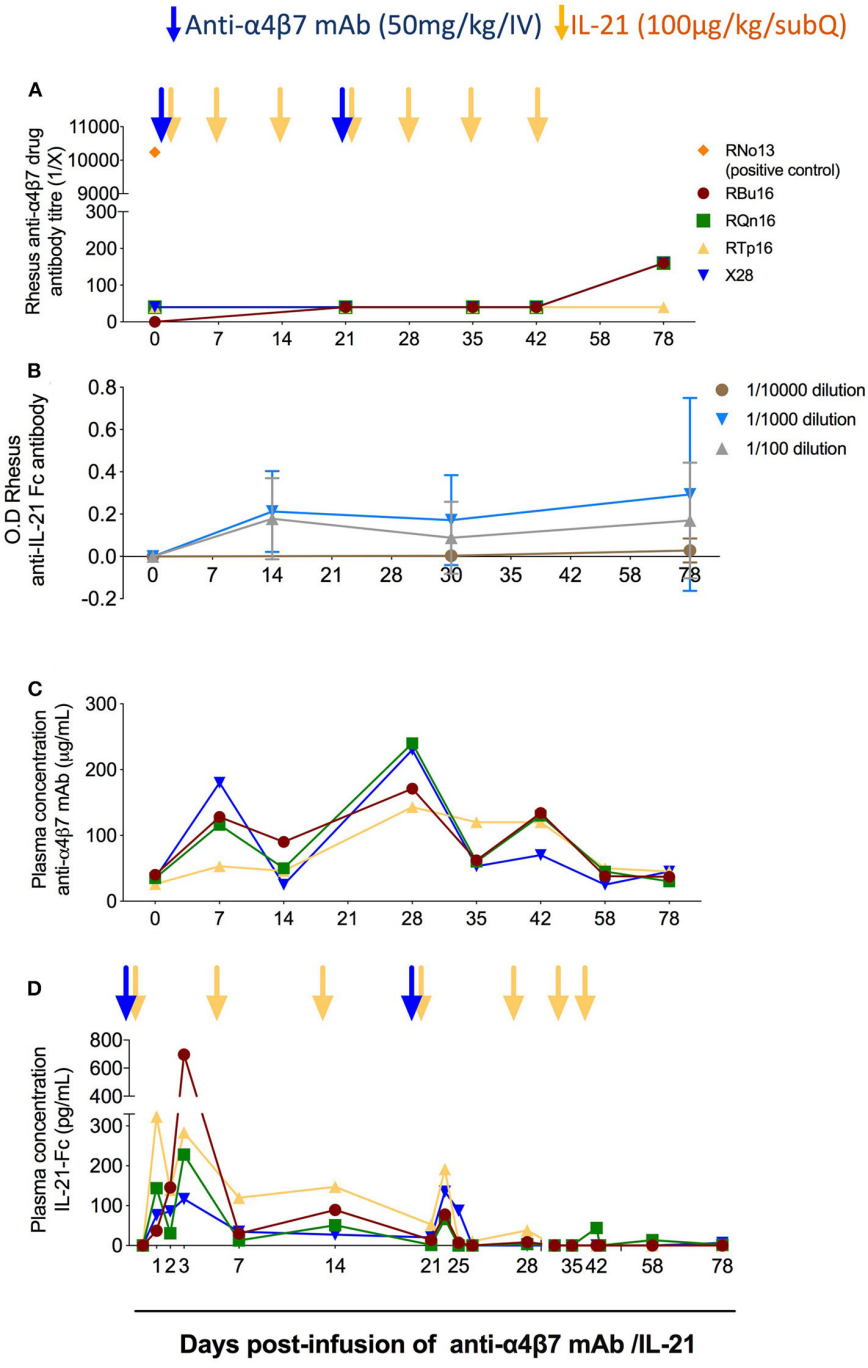
Co-administration of anti-α4β7 mAb and IL-21 does not elicit reactive antibodies**. (A)** Analysis of rhesus antibodies anti-α4β7 mAb in plasma at different time points. The anti-drug antibodies levels were measured by ELISA end point titer method as described in the Materials and Methods section. RNo13, indicated in orange, corresponds to the plasma of an animal from a previous study that developed ADAs (21), and it was used as a positive control. **(B)** Measurement of anti–IL-21–Fc antibodies in plasma at different time points. Anti–IL-21–Fc antibodies were measured by ELISA longitudinally until the end of the study with three different dilutions (1:100, 1:1,000, and 1:10,000). **(C)** Measurement of anti-α4β7 mAb plasma levels (μg/mL) in RMs (*n* = 4). The assay was performed using flow cytometry with HuT 78 cells. The levels of anti-α4β7 mAb were measured employing standard curve method. Mean fluorescence intensity (MFI) of known concentration of anti-α4β7 mAb was obtained, and then the MFI of plasma levels of anti-α4β7 mAb was plotted. **(D)** Measurement of IL-21–Fc plasma levels (pg/mL) in RMs (*n* = 4). ELISA background and plasma baseline values were subtracted from the values analyzed at each time point. Individual animals are represented with different colors and symbols. Blue arrows indicate the anti-α4β7 mAb intravenous infusions, and yellow arrows indicate IL-21 subcutaneous infusions.

Previous studies have shown that the administration of anti-α4β7 mAb can lead to the development of ADA in a subset of RMs, which resulted in loss of anti-α4β7 mAb biological activity (21, 23, 24). In one of those studies, in which 11 RMs received eight intravenously doses of the anti-α4β7 mAb (50 mg/kg each; at weeks 9, 12, 16, 18, 20, 24, 28, and 32 post-SIV infection), three animals developed ADA responses starting either after two, three, or six doses (21). In order to test whether repeated and combined infusions of IL-21 and anti-α4β7 mAb induced ADA responses, we measured the levels of rhesus ADA against the anti-α4β7 mAb in the plasma of the four treated RMs. The plasma end point titers for all RMs before infusion as well as after infusion and until day 78 remained unchanged (Figure 2A). A positive control serum from monkey RNo13 was used as a positive control, which was collected during the aforementioned *in vivo* study (21), with a titer of 1:10,000 (Figure 2A). Similarly, we did not find any measurable levels of anti–IL-21–Fc in the plasma of the four RMs neither at any tested time points or at any tested dilutions (1:100, 1:1,000, and 1:10,000) (Figure 2B), whereas positive control showed measurable anti–IL-21–Fc levels, confirming the correct coating of the plate (data not shown). These results indicate that, at least under the conditions used in this study, the co-administration of IL-21 and anti-α4β7 mAb did not promote the induction of ADA against either therapeutic agent. Next, we quantified the levels of anti-α4β7 mAb in plasma using flow cytometry as described in section Materials and Methods and as previously published (20). Mean baseline levels of anti-α4β7 mAb in all RMs before anti-α4β7 mAb administration were less than 40 μg/mL (Figure 2C); this is likely due to pre-existing antibodies against anti-α4β7 or assay background. The mean plasma levels of anti-α4β7 mAb increased to 95.6 μg/mL and to 213 μg/mL by day 7 after the first and second dose of 50 mg/kg infusion of anti-α4β7 mAb, respectively; the mean plasma levels were maintained at 92 μg/mL until day 42 post-infusion (Figure 2C). The stable levels of anti-α4β7 mAb during weekly administration of IL-21 suggest that IL-21 did not markedly influence levels of the anti-α4β7 mAb and that there were no drug–drug interactions. Finally, we quantified IL-21–Fc levels in plasma by ELISA. IL-21–Fc plasma levels increased after IL-21 infusion for all four animals compared with their baseline levels (<15.625 pg/mL in all animals). Its maximum concentration was achieved at day 1 post-infusion for RTp16 (323.24 pg/mL), and day 3 post-infusion for the remaining animals (RBu16: 696.54 pg/mL, RQn16: 228.3 pg/mL, and X28: 116.6 pg/mL) (Figure 2D). Increased plasma levels of IL-21 were still evident on day 22, 1 day after the fourth IL-21 infusion (performed on day 21), but attenuated afterward.

### The Combined IL-21 and Anti-α4β7 mAb Treatment Reduces Gut Homing of Memory CD4 T Cells Expressing α4β7

**Figure 3:**
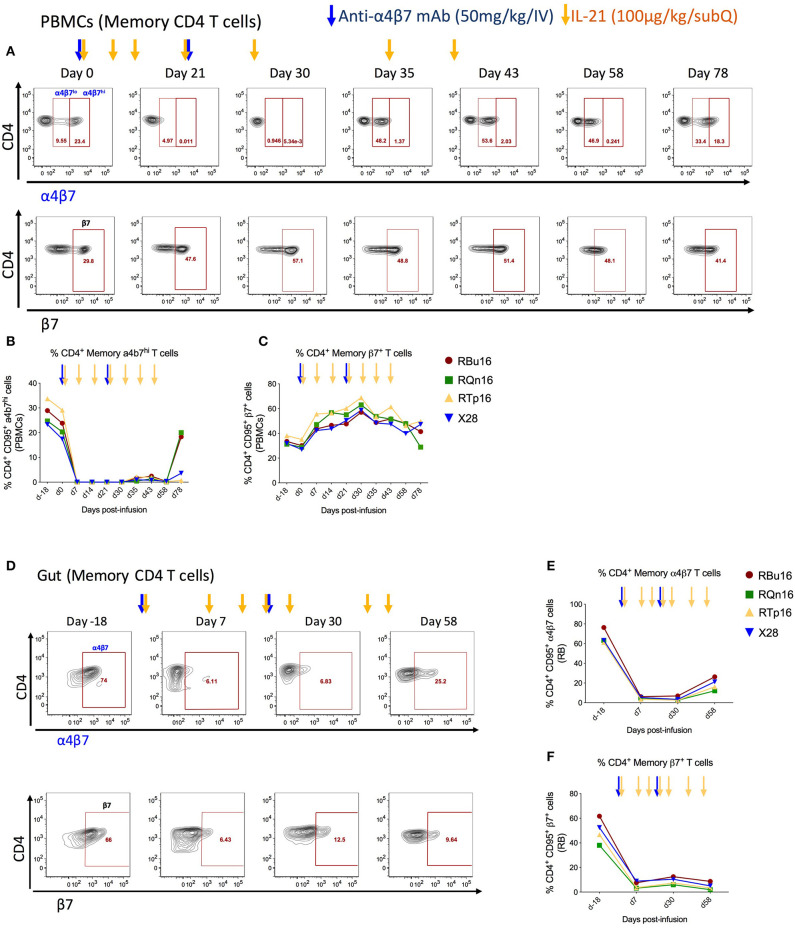
Co-administration of anti-α4β7 mAb and IL-21 decreases gut homing of CD4 α4β7^hi^ to gut. **(A)** Representative flow cytometry staining of memory CD4 T cells expressing α4β7^hi^ (top panel) and β7^+^ (bottom panels) in PBMCs. **(B)** Frequency of α4β7^hi^ and **(C)** β7^+^ memory CD4 T cells in PBMCs. **(D)** Representative flow cytometry staining of memory CD4 T cells expressing α4β7^+^ (top panel) and β7^+^ (bottom panels) in gut. **(E)** Frequency of α4β7^+^ and **(F)** β7^+^ memory CD4 T cells in gut. Individual animals are represented with different colors and symbols. Baseline days are indicated as d-18 and d0. Blue arrows indicate the anti-α4β7 mAb intravenous infusions, and yellow arrows indicate IL-21 subcutaneous infusions.

To investigate the impact of combined IL-21 and anti-α4β7 mAb therapy impact on T-cell gut homing, we quantified the frequency of memory CD4 (CD3^+^CD4^+^CD95^+^) and CD8 (CD3^+^CD8^+^CD95^+^) T cells expressing α4β7^hi^ or β7 in blood (Figures 3A–C, and Supplementary Figures 1A–C) and RBs (Figures 3D–F and Supplementary Figures 1D–F) collected longitudinally during the study. The frequencies of α4β7^hi^ CD4 memory T cells decreased by greater than 99% in blood (Figure 3B) and 92.5% in RBs (Figure 3E) already at 1 week after the first anti-α4β7 mAb infusion; these measured levels remained constant up to day 58 post-infusion, ~5 weeks after the second dose of anti-α4β7 mAb and gradually increased thereafter, although they remained still below baseline on day 78 post-infusion, the latest time point of the study (Figures 3B,E). Similar results were found for CD8 T cells, with the frequencies of α4β7^hi^ CD8 memory T cells decreased by greater than 99% in blood (Supplementary Figure 1B) and 97% in RB (Supplementary Figure 1E). Because the mAb used for flow cytometry staining recognizes the same epitope as the anti-α4β7 mAb used *in vivo*, these data indicate the biological activity of the anti-α4β7 mAb in targeting α4β7 expressed on CD4 and CD8 T cells in blood and gut. Of note, the very low frequencies of α4β7^hi^ CD4 and CD8 memory T cells were maintained during the IL-21 only administrations (days 30, 35, and 43 post-infusion) that followed the last dose of anti-α4β7 mAb (day 21 post-infusion), showing that the administration of IL-21 did not influence the expression of α4β7 or the ability of the anti-α4β7 mAb to target it. Furthermore, PBMCs were monitored for the expression of β7 using an anti-β7 mAb that does not compete for the epitope recognized by the anti-α4β7 mAb administered *in vivo*; as such, this analysis allows for discriminating whether the inability to stain for α4β7 results from *in vivo* depletion of α4β7 expressing cells or masking of the α4β7 molecule on cells, as well as to determine the impact of the treatment on the trafficking of β7^+^ cells to the gut. As shown in Figure 3C, β7 expression on blood memory CD4 T cells was increased up to 2-fold at day 30 post-infusion as compared to pre-treatment levels (Figure 3C; from 30.25 to 61.88%). In RBs, frequencies of β7^+^ cells were significantly lower up to day 58 post-infusion as compared to pre-treatment (Figure 3F, from 49.7 to 4.74%). Interestingly, β7 expression on memory CD8 T cells differed from that of memory CD4 T cells after anti-α4β7 mAb administration. Specifically, frequencies of memory CD8 T cells expressing β7 were reduced from 38.7 to 10.77% in blood, whereas they remained stable in the gut (Supplementary Figures 1C, F). Together, these data indicate that anti-α4β7 mAb treatment combined with IL-21 effectively reduces the homing of α4β7^hi^ memory CD4 T cells to the gut mucosa.

### Effect of Combined IL-21 and Anti-α4β7 mAb Treatment on NK Cells

We then measured the effect of combined IL-21 and anti-α4β7 mAb treatment on the frequency (in PBMCs and RB) and absolute number (limited to PBMCs) of NK cells and NK cell subsets (CD56^−^CD16^+^, CD56^+^CD16^−^, and CD16^−^CD56^−^). Overall, the levels of NK cells and their subsets remained stable during the treatment both in PBMCs and RB. A slight increase was noted in the frequency (of total lymphocytes) of CD56^+^CD16^−^ and CD56^−^CD16^−^ NK cells in PBMCs during the treatment (Supplementary Figures 2A,B). In RB, increased frequency (of live cells) of NK cells was observed between days 7 and 30, followed by reduction to baseline levels upon interruption of IL-21 therapy (d58; Supplementary Figure 2C). This slight increase of bulk NK cells resulted in an increased frequency of CD56^−^CD16^+^ from baseline (d-18) to day 30 (Supplementary Figure 2D) and of CD56^+^CD16^−^ from baseline to day 7 (Supplementary Figure 2E), which return to baseline levels at day 58. In RB, CD56^−^CD16^−^ NK cells remained constant across the course of the study and were not affected by withdrawal of IL-21 therapy (not shown).

### Combined IL-21 and Anti-α4β7 mAb Treatment Limits Immune Activation and Cell Cycling of Gut Memory CD4 T Cells

**Figure 4:**
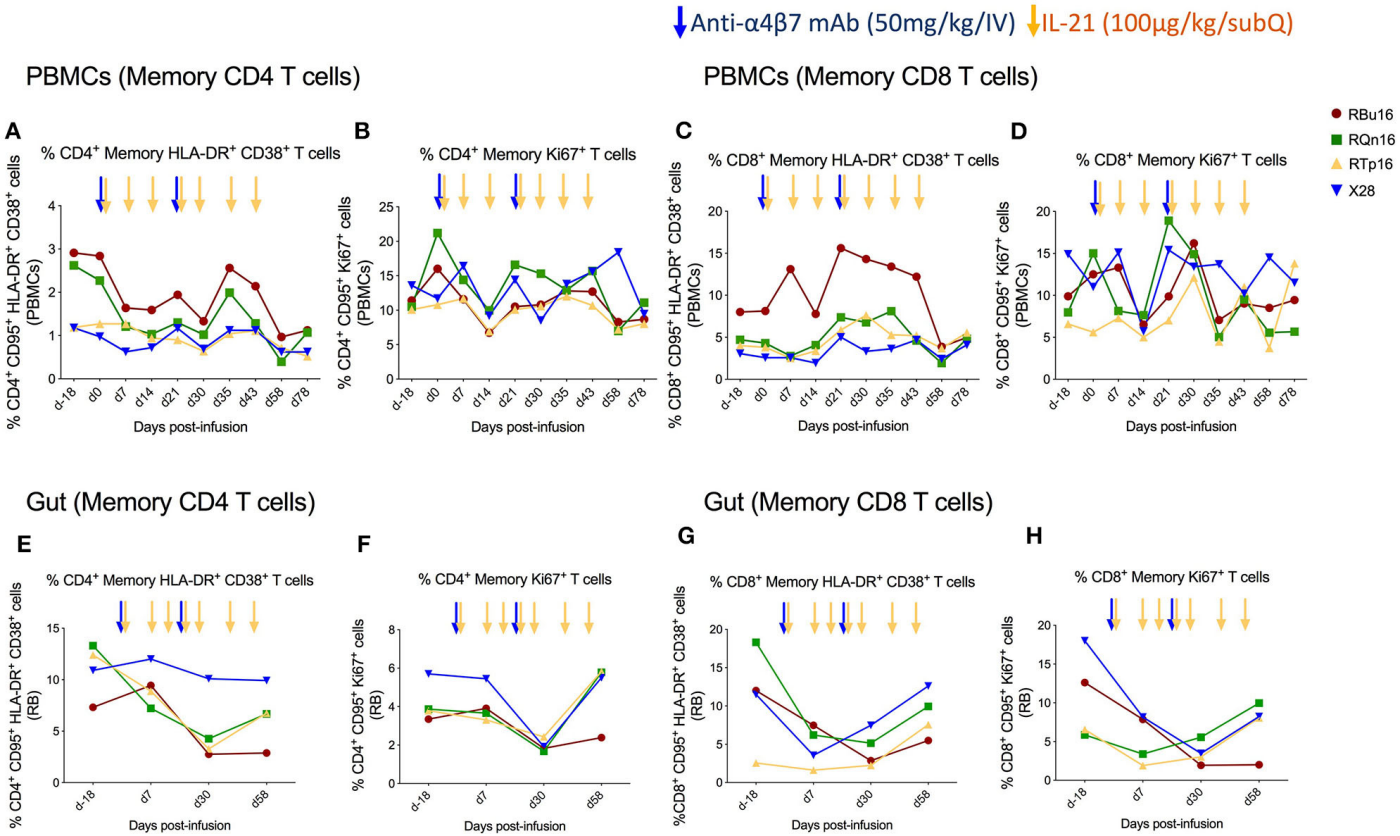
Co-administration of anti-α4β7 mAb and IL-21 limits immune activation and cell cycling of memory CD4 T cells in gut**. (A)** Percentage of memory CD4 T cells expressing HLA-DR^+^CD38^+^, **(B)** Ki67^+^, **(C)** memory CD8 T cells expressing HLA-DR^+^CD38^+^, and **(D)** Ki67^+^ in PBMCs. **(E)** Percentage of memory CD4 T cells expressing HLA-DR^+^CD38^+^, **(F)** Ki67^+^, **(G)** memory CD8 T cells expressing HLA-DR^+^CD38^+^, and **(H)** Ki67^+^ in gut mucosa. Individual animals are represented with different colors and symbols. Baseline days are indicated as d-18 and d0. Blue arrows indicate the anti-α4β7 mAb intravenous infusions, and yellow arrows indicate IL-21 subcutaneous infusions.

We assessed the effects of the combined IL-21 and anti-α4β7 mAb therapy on systemic and gut immune activation. For this aim, we measured the frequency of memory T cells expressing markers of activation (HLA-DR and CD38) and cell cycling (Ki67). The frequency of blood memory CD4 and CD8 T cells with an HLA-DR^+^CD38^+^ (Figures 4A,C) or Ki67^+^ (Figures 4B,D) phenotype remained similar overall, with a slight decrease at specific time points. Specifically, the frequency of HLA-DR^+^CD38^+^ T cells was lower as compared to baseline on days 30, 58, and 78 after IL-21 plus anti-α4β7 mAb treatments for memory CD4 and at day 58 post-treatment for memory CD8 T cells. The frequency of Ki67^+^ T cells was lower as compared to baseline only at day 14 post-treatment both for memory CD4 and CD8 T cells. Importantly, differences were more pronounced in gut, with a progressive reduction in the frequency of memory CD4 and CD8 T cells that are HLA-DR^+^CD38^+^ (Figures 4E,G) or Ki67^+^ (Figures 4F,H) from baseline to day 30 post-treatment. The reduction in both immune activation and cell cycling in the gut is consistent with CD4 T cells expressing α4β7^hi^ being retained in blood as a result of the combined treatment. Collectively, our data show that a strategy based on the combined administration of IL-21 and anti-α4β7 mAb is effective in blocking the homing of memory CD4^+^ α4β7^hi^ T cells to the gut and in reducing mucosal immune activation, even in healthy, SIV-uninfected RMs.

## Discussion

The results of the present study indicate that treatment of healthy, SIV-uninfected RMs with a combined IL-21 and anti-α4β7 mAb intervention (1) is safe and well-tolerated (at the tested doses), (2) does not affect bioavailability of both compounds, (3) can effectively bind the α4β7 receptor on both blood and gut mucosa T cells, and (4) reduces cell cycling and immune activation, particularly in gut mucosa. The data generated in this pilot study support future combined interventions in ART-treated, SIV-infected non-human primates, aimed at limiting residual inflammation and viral persistence, particularly in the gut mucosa.

At the doses administered in our study, combined administration of IL-21 and anti-α4β7 mAb was well-tolerated and did not result in any detectable toxicity. These results are aligned with previous studies where IL-21 and anti-α4β7 mAb were tested independently and proved to be safe in SIV-infected RMs (9, 10, 21). IL-21 treatment in RMs can increase the JAK/STAT signaling pathway, implicated to have roles in inflammation processes (10), whereas gene expression studies in vedolizumab-treated patients revealed dysregulated expression of genes related to cell cycle, cell growth, and inflammation (26). In our study, the combined treatment was not associated with any increase in parameters related to inflammation or immune activation.

The production of antibodies against drugs administered *in vivo* not only can reduce the bioavailability and biological activity of the administered compounds, but can also elicit the development of immune-mediated adverse events. In our study, we have not observed development of ADA responses against anti-α4β7 mAb or IL-21. In previous studies, which used anti-α4β7 mAb in SIV-infected RMs at similar doses as our study (500 μg/kg), a fraction of the animals developed ADA starting from the second, third, or sixth infusion (Byrareddy et al., 3 of 11 animals; Di Mascio et al., 1 of 12 animals). Our pilot study suggests that IL-21 does not favor the generation of ADA against anti-α4β7 mAb. Otherwise, we have not been able to detect antibodies against IL-21–Fc, despite we found a reduced plasma concentration of IL-21 after the fourth dose. To date, we have not seen development of ADA in any of the RMs we treated with a similar dose of IL-21 in the past several years (9, 10). It is possible that inhibition of α4β7 using anti-α4β7 mAb contributed to decreased levels of IL-21 and/or that repeated dosage of IL-21 resulted in saturation or decreased expression of the IL-21 receptor, making IL-21 to be freely available for a faster clearance as compared to bound IL-21. The consistent increase of plasma concentration of anti-α4β7 mAb during IL-21 infusions indicates that IL-21 does not negatively affect anti-α4β7 mAb bioavailability. Interestingly, there is an unexpected increase of anti-α4β7 plasma levels at day 42, present in three of the four treated RMs, without any new anti-α4β7 mAb administration. One possibility is that this results from differences in receptor activation (27) and or recycling processes, as observed for β1 integrin receptor in a previous study (28), altering the number of receptors able to bind the administered anti-α4β7 Ab. It is also possible that IL-21 administration contributed to increase plasma levels of anti-α4β7, although this cannot be directly proved in our pilot.

Of note, the anti-α4β7 antibodies used for staining and infusing the animals bind to the same antigen; thus, a lack of α4β7 staining by flow cytometry is interpreted as a measure of targeting engagement, that is, ability of the inoculated antibody to bind α4β7 expressed on cell surface, without discriminating if the lack of staining is due to receptor downregulation, cell lysis, or receptor blockage by the competing antibody. Interestingly, we discovered an increase in the frequency of β7^+^ CD4 memory T cells in blood, but a decrease of these cells in RB. These findings suggest that the administration of anti-α4β7 mAb reduced the trafficking of α4β7^hi^ T cells to the gut, confirming the mode of action, prevention of trafficking of activated T cells to the gut, of vedoluzimab as adjunctive therapy in inflammatory bowel disease and Crohn disease (IBD/CD) (29–34). As such, a similar strategy is of interest in the context of HIV infection, where CD4 T cells that express CCR5 and α4β7 are the preferred target for HIV infection in the gut, a major site for early HIV infection and replication (2, 35–37).

Combined administration of IL-21 and anti-α4β7 antibody decreased the frequency of T cells expressing immune activation and proliferation markers in the gut of healthy RMs, despite the low baseline level. This result supports the use of this combined treatment in the context of SIV infection in RMs. Our previous studies showed that, by favoring maintenance of T_H_17 and T_H_22 cells, IL-21 improves mucosal integrity and reduces inflammation when administered in acute (9) or chronic, ART-treated (10) SIV-infected RMs. Similarly, anti-α4β7 antibody reduced SIV infection in the GALT when animals were challenged either intravenously, intrarectally, and intravaginally (18, 38, 39). In another recent study, the combination of primatized anti-α4β7 and VRC01 significantly delayed vaginal SHIV exposure and reduced viral loads in rectal tissues compared to control (40). The effect of anti-α4β7 mAb administered in SIV-infected RMs during ART continues to be a highly debatable issue. While an earlier study showed that this treatment can limit viral rebound after ART interruption (21), more recent pre-clinical (23–25) and clinical (41) studies did not show any significant benefit from anti-α4β7 mAb treatment in ART-suppressed, HIV-infected individuals or SIV-infected RMs in inducing viral remission in the absence of ART (42). Recently, using samples obtained from various gastrointestinal sites from IBD/CD patients, it was found that anti-α4β7 therapy led to a significant reduction of lymphoid aggregates, mostly in the terminal ileum (43). Because lymphoid aggregates serve as important sanctuary sites for maintaining viral reservoirs, the authors proposed that their ablation by anti-α4β7 mAb should be considered in developing novel therapies for HIV remission. These findings highlight that much has yet to be learned about the mechanisms of action and biologic effects of anti-α4β7 therapy, as well as on the combination of anti-α4β7 mAb with additional immunotherapies to provide immunologic and virologic benefits.

This is the first study in non-human primates showing that anti-α4β7 mAb and IL-21 treatment can be administered safely and can reduce cell cycling and immune activation, particularly in gut mucosa. As such, our study provides rationale to explore this combined treatment as a strategy aimed at limiting immune activation and viral persistence in ART-suppressed, SIV-infected RMs.

## Data Availability Statement

All datasets generated for this study are included in the article/Supplementary Material.

## Ethics Statement

The animal study was reviewed and approved by Institutional Animal Care and Usage Committees at the Yerkes National Primate Research Center, Emory University.

## Author Contributions

MPi, MPa, and SB contributed to study design. MPi, SU, KP, CK, KN, IS, and KR contributed to data collection. MPi, SU, KP, KR, and SB contributed to data quality and analysis. MPi, SU, FV, MPa, and SB wrote the manuscript. All authors contributed to manuscript development and have critically reviewed and approved the final version.

## Conflict of Interest

The authors declare that the research was conducted in the absence of any commercial or financial relationships that could be construed as a potential conflict of interest.
