# Supplementary material for: Safety and Immunological Evaluation of Interleukin-21 Plus Anti-α4β7 mAb Combination Therapy in Rhesus Macaques
Source: Front Immunol. 2020 Jul 17;11:1275. doi: 10.3389/fimmu.2020.01275 (PMC7379916; doi:10.3389/fimmu.2020.01275)
Supplement: Supplementary file 1 [file Table_1.docx]

**Supplementary Table 1. Characteristics of RMs used in this study.**

|  | | | **MHC genotype** | | |
| --- | --- | --- | --- | --- | --- |
| **Animal ID** | **Sex** | **Age** | **A01** | **B08** | **B17** |
| RBu16 | Male | 3 years 3 months | - | - | - |
| RQn16 | Male | 3 years 2 month | - | - | - |
| RTp16 | Female | 3 years 2 month | - | - | - |
| X28 | Female | 3 years 2 month | - | - | - |
