# Supplementary material for: Safety and Immunological Evaluation of Interleukin-21 Plus Anti-α4β7 mAb Combination Therapy in Rhesus Macaques
Source: Front Immunol. 2020 Jul 17;11:1275. doi: 10.3389/fimmu.2020.01275 (PMC7379916; doi:10.3389/fimmu.2020.01275)
Supplement: Supplementary file 2 [file Table_2.docx]

**Supplementary Table 2. Antibodies used in flow cytometry analysis.**
