# Supplementary figures and images for: Safety and Immunological Evaluation of Interleukin-21 Plus Anti-α4β7 mAb Combination Therapy in Rhesus Macaques
Source: Front Immunol. 2020 Jul 17;11:1275. doi: 10.3389/fimmu.2020.01275 (PMC7379916; doi:10.3389/fimmu.2020.01275)

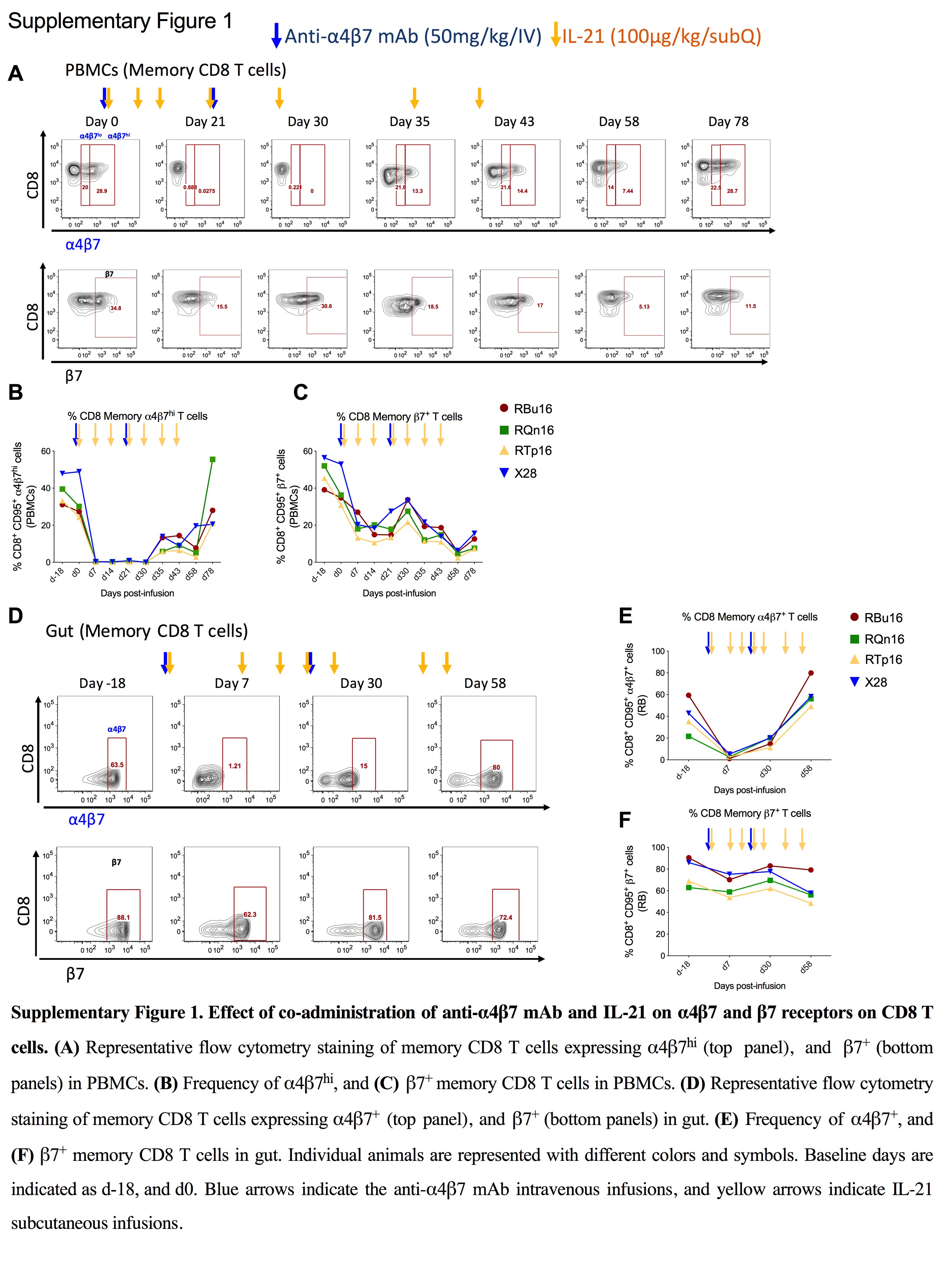

Supplement: Supplementary file 3 [file Image_1.jpg]

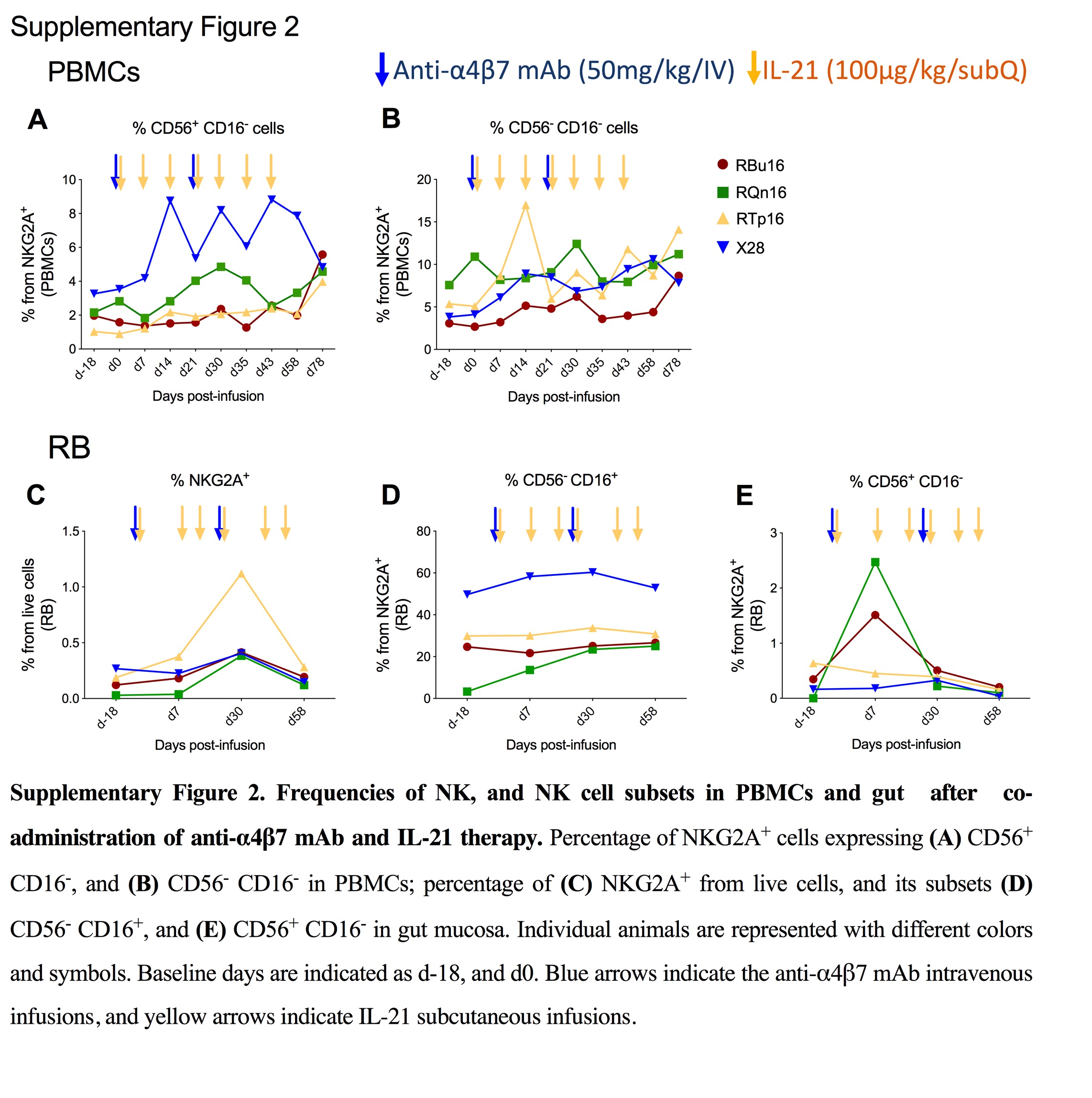

Supplement: Supplementary file 4 [file Image_2.jpg]
